# Robust tests for matched case-control genetic association studies

**DOI:** 10.1186/1471-2156-11-91

**Published:** 2010-10-12

**Authors:** Yong Zang, Wing Kam Fung

**Affiliations:** 1 Department of Statistics and Actuarial Science, The University of Hong Kong, Hong Kong, China

## Abstract

**Background:**

The Cochran-Armitage trend test (CATT) is powerful in detecting association between a susceptible marker and a disease. This test, however, may suffer from a substantial loss of power when the underlying genetic model is unknown and incorrectly specified. Thus, it is useful to derive tests obtaining the plausible power against all common genetic models. For this purpose, the genetic model selection (GMS) and genetic model exclusion (GME) methods were proposed recently. Simulation results showed that GMS and GME can obtain the plausible power against three common genetic models while the overall type I error is well controlled.

**Results:**

Although GMS and GME are powerful statistically, they could be seriously affected by known confounding factors such as gender, age and race. Therefore, in this paper, via comparing the difference of Hardy-Weinberg disequilibrium coefficients between the cases and the controls within each sub-population, we propose the stratified genetic model selection (SGMS) and exclusion (SGME) methods which could eliminate the effect of confounding factors by adopting a matching framework. Our goal in this paper is to investigate the robustness of the proposed statistics and compare them with other commonly used efficiency robust tests such as MAX3 and *χ*^2 ^with 2 degrees of freedom (df) test in matched case-control association designs through simulation studies.

**Conclusion:**

Simulation results showed that if the mean genetic effect of the heterozygous genotype is between those of the two homozygous genotypes, then the proposed tests and MAX3 are preferred. Otherwise, *χ*^2 ^with 2 df test may be used. To illustrate the robust procedures, the proposed tests are applied to a real matched pair case-control etiologic study of sarcoidosis.

## Background

The population-based case-control association study is a powerful approach in detecting the association between a candidate marker and a disease. Compared with the family-based association study which recruits samples from family members, the case-control study is more cost effective because cases and controls are unrelated hence easy to recruit from population. To test the genetic association using the case-control design, the genotypic data for a bi-allelic marker are usually described by a 2 × 3 table where rows represent the disease status and columns represent the genotypic counts. Hence, to test for genetic association is equivalent to test for association between the rows and the columns. Generally, the Pearson's *χ*^2 ^with 2 df test can be used to detect such an association. Besides, if a linear trend among the rows can be assumed, a more powerful test which utilizes the score test for a logistic regression can be obtained. This score test is known as the Cochran-Armitage trend test (CATT)[1-3].

To apply the CATT, increasing scores are specified a priori for the underlying genetic model. A genetic model refers to the model of inheritance, which defines some relationship of the risks of having the disease given different genotypes. The common genetic models include, but not limit to, recessive (REC), additive (ADD) and dominant (DOM) models. If the underlying genetic model is known, the asymptotically optimal CATT can be used. Otherwise, the CATT is not robust when the scores are misspecified [4]. Unfortunately, the underlying genetic model is usually unknown in practice and an incorrect choice of the genetic model may result in a substantial loss of power for the CATT. Thus, a robust method which does not assume a prior knowledge of the underlying genetic model is often useful.

Methods robust for a variety of underlying model of inheritance have recently become an important area of research. The Pearson's *χ*^2 ^test with 2 df does not assume any structure of a genetic model so it is a robust test against the genetic model. Moreover, the maximin efficiency robust test (MERT) and the MAX method using the maximum of the CATTs optimal for REC, ADD and DOM respectively were extensively studied [5-10]. Recently, Zheng and Ng [11] proposed the genetic model selection (GMS) method to test for genetic association. Different from other robust tests, the GMS approach is a two-phase analysis which uses the Hardy-Weinberg disequilibrium trend test (HWDTT) [12] to choose the most suitable genetic model in the first phase followed by the CATT optimal for the selected genetic model to detect the association in the second phase. Since the same data were used twice in the analysis, the nominal type I error for the second test needs to be adjusted so that the GMS can obtain a correct size. This GMS has an assumption that the marker allele associated with the disease allele is known. Such assumption can be difficult to justify, for instance, in many complex diseases. Thus, to remove this restriction, Joo et al. [13] proposed to use the CATT optimal for the ADD model to detect the risk allele. After the risk allele is determined, the GMS corresponding to the detected risk allele is then carried out. As a result, using their GMS, people do not need to assume that the risk allele is known. Besides the modified GMS, Joo et al. [13] developed another two-phase test called the genetic model exclusion (GME) which excludes the most unlikely genetic model rather than selecting the most likely one. They also showed that when the genetic relative risks (GRRs) are small, the GME is more efficiency robust than the GMS. Besides the frequentist analysis, a Bayesian hierarchical model which regards the genetic model parameter as a fixed effect has been proposed by Minelli et al. [14]. If expert opinion or external evidence is available, an informative prior distribution of the genetic model parameter could be adopted; otherwise, a vague prior distribution should be used to avoid the undue influence on the posterior distribution.

Although the population based case-control study is powerful and feasible to implement, spurious association may arise due to known confounding factors such as gender, age and race. Intuitively, the GMS and GME do not work in the presence of confounding factors. One of the reasons is that when the samples are divided into several sub-populations via the confounding factors, the Hardy-Weinberg equilibrium (HWE) assumption needed in the first phase of the GMS and GME does not hold any more. Besides, the CATTs used in the second phase of the GMS and GME do not control the size well due to the confounding factors.

Typically, when the confounding factors can be observed, they could be treated as the covariates of interest and incorporated in the logistic regression. However, further calculation to adjust for the covariates may complicate the trend test. Alternatively, the matching strategy is frequently used as a much simpler way to control potential confounding factors in epidemiological studies. Specifically, a single case is matched with a certain number of controls based on the confounding factors constructing for each matched set. Then, a conditional logistic regression analysis is normally used to fit the matched data. Recently, an increasing number of matching studies are conducted by either adopting the matched design [15-18] or developing statistical procedures [19-24] for matched genetic association studies.

Similar to the unmatched case-control association study, when the underlying genetic model is unknown, the robustness of the statistics for the matched case-control design is also worth studying. Zheng and Tian [21] proposed the MAX3 test based on the matching trend test (MTT) derived from a conditional logistic regression. However, to our knowledge, this is the only paper discussing the robust tests in matched case-control design, compared to the large amount of literature in the unmatched design. Thus, in this paper, we start by developing the stratified genetic model selection (SGMS) and exclusion (SGME) methods for matched case-control association, then we study the robustness of the test statistics. The performance of the robust tests and MTTs is compared by simulation for a wide range of scenarios. Finally, the tests are applied to a real matched pair case-control etiologic study of sarcoidosis.

## Methods

### Genetic model selection and exclusion

When the genetic model is unknown, the *T*_HWDTT _test proposed by Song and Elston [12] can be used to detect the latent genetic model. Zheng and Ng [11] demonstrated that under Hardy-Weinberg equilibrium (HWE) and when the allele investigated is the risk allele (denoted as *D*), *T*_HWDTT _> 0 under the REC model and *T*_HWDTT _< 0 under the DOM model. Denote *T*_0_, *T*_0.5 _and *T*_1 _as the CATTs optimal for REC, ADD and DOM respectively, Zheng and Ng [11] proposed to use *T*_0 _if *T*_HWDTT _>*c*; *T*_1 _if *T*_HWDTT _< -*c *and *T*_0.5 _otherwise to test for genetic association, where *c *is a pre-specified threshold.

Note that for the original GMS mentioned above, the risk allele is assumed to be known. However, if the risk allele cannot be correctly specified, such GMS may have some problems. Specifically, consider a bi-allelic marker with alleles *D *and *d *and assume *D *is the risk allele. So if *D *is really the risk allele, *T*_0_, *T*_0.5 _and *T*_1 _are optimal for the REC, ADD and DOM models respectively. On the other hand, if *d *is the true risk allele, then -*T*_1_, -*T*_0.5 _and -*T*_0 _are optimal for the REC, ADD and DOM models respectively. Joo et al. [13] proposed to use *T*_0.5 _to decide which one is the risk allele followed by the corresponding GMS which depends on the determined risk allele. Joo et al. [13] suggested the modified GMS which can be written as

(1) TGMS=T0I(T0.5>0)I(THWDTT>c)+T0.5I(T0.5>0)I(|THWDTT|≤c)+T1I(T0.5>0)I(THWDTT <−c)−T1I(T0.5≤0)I(THWDTT>c)−T0.5I(T0.5≤0)I(|THWDTT|≤c)−T0I(T0.5≤0)I(THWDTT <−c),

where I(.) is an indicator function.

When the GRRs are small, Joo et al. [13] found that the probability of selecting the true genetic model by using *T*_HWDTT _becomes small. On the other hand, the probability of correctly excluding the most unlikely genetic model remains high against the GRRs. Furthermore, when the most unlikely genetic model is excluded, the simple MERT [5] can be carried out to build a robust test against the remaining models. These facts inspired Joo et al. [13] to develop the genetic model exclusion (GME) approach. Specifically, first denote $T_{0}^{*}=(T_{0}+T_{0.5})/(\sqrt{2(1+{\hat{\rho }}_{0,0.5})}),T_{0.5}^{*}=T_{0.5}$ and $T_{1}^{*}=(T_{1}+T_{0.5})/(\sqrt{2(1+{\hat{\rho }}_{1,0.5})})$ where ${\hat{\rho }}_{x_{1},x_{2}}$ is an estimate of the correlation between $T_{x_{1}}$ and $T_{x_{2}}$ under the null hypothesis of no association, then one can obtain the GME statistic from the GMS test by replacing *T*_0_, *T*_0.5 _and *T*_1 _in (1) by $T_{0}^{*}$, $T_{0.5}^{*}$ and $T_{1}^{*}$ respectively. Since the GMS and GME are two stage tests and the same data set is used twice, the critical values of the tests in the second stage need to be adjusted to control the overall type I error rates; see Zheng and Ng [11] and Joo et al. [13].

Although GMS and GME are efficiency robust tests, they could be seriously affected by confounding factors. In the presence of sub-populations, GMS and GME may not keep the correct size. Therefore, to overcome this limitation, we propose the stratified genetic model selection (SGMS) and exclusion (SGME) approaches in the following.

### Notation

Consider a bi-allelic marker with alleles *d *and *D *and assume *D *is the risk allele. Denote the three genotypes of this marker as *G*_0 _= *dd*, *G*_1 _= *Dd *and *G*_2 _= *DD*. Suppose that the confounding factors define *L *strata, denoted by *C_l_*, *l *= 1,..., *L*. In the *l*th stratum, *r*_l _cases are drawn from the population and *m *controls are matched to each case. Thus, the total number of controls in the *l*th stratum is *s_l _*= *mr_l _*for *l *= 1,..., *L*. The genotype counts for (*G*_0_, *G*_1_, *G*_2_) in cases and controls in the *l*th stratum are denoted by (*r*_0*l*_, *r*_1*l*_, *r*_2*l*_) and (*s*_0*l*_, *s*_1*l*_, *s*_2*l*_), respectively. Hence, $r_{l}=\sum_{i=0}^{2}r_{il}$ and $s_{l}=\sum_{i=0}^{2}s_{il}$. The total number of cases is *r *= ∑*_l _r_l _*and the total number of controls is *s *= *mr*. The total sample size is then *n *= (*m *+ 1)*r*.

In the *l*th stratum (*l *= 1,..., *L*), denote the penetrance by *f_il _*= *Pr*(case|*G_i_*, *C_l_*) for *i *= 0, 1, 2, the disease prevalence by *k_l _*= *Pr*(case|*C_l_*) = ∑*_i _f_il_Pr*(*G_i_|C_l_*), and the genotype frequencies in cases and controls by *p_il _*= *Pr*(*G_i_|*case,*C_l_*) = *f_il_Pr*(*G_i_|C_l_*)/*k_il _*and *q_il _*= *Pr*(*G_i_|*control, *C_l_*) = (1 - *f_il_*)*Pr*(*G_i_|C_l_*) = (1 - *k_il_*), respectively. Define GRRs in the *l*th stratum as *λ*_1*l *_= *f*_1*l*_/*f*_0*l *_and *λ*_2*l *_= *f*_2*l*_/*f*_0*l *_(*f*_0*l *_> 0). A genetic model is REC, ADD and DOM if *λ*_1*l *_= 1, *λ*_1*l *_= (*λ*_2*l *_+ 1)/2 and *λ*_1*l *_= *λ*_2*l*_, respectively. We assume that HWE holds in each stratum. Thus, $Pr(G_{0}|C_{l})=q_{l}^{2}$, Pr(*G*_1_|*C*_l_) = 2*p_l_q_l _*and $Pr(G_{2}|C_{l})=p_{l}^{2}$ where *p_l _*is the allele frequency of *A *in the *l*th stratum and *q_l _*= 1 - *p_l_*.

### Stratified genetic model selection and exclusion

Let *X*_1*lj *_and *X*_2*ljk *_denote the genotypic scores for the *j*th case and the *k*th control matched with the *j*th case in the *l*th stratum, *j *= 1,..., *r_l_*, *k *= 1,..., *m *and *l *= 1,..., *L*. Each score takes one of the three possible values: 0, *x *or 1 for the genotypes *G*_0_, *G*_1 _or *G*_2 _respectively, where *x *is 0, 0.5 or 1 for the REC, ADD or DOM model. Following Day and Byar [25] and Zheng and Tian [21], the likelihood function conditional on the outcomes of cases and matched controls for the candidate marker can be written as

(2) L(β|G,C1,…,CL)=∏l=1L∏j=1rlexp(αl+βX1lj)exp(αl+βX1lj)+∑k=1mexp(αl+βX2ljk)=∏l=1L∏j=1rlexp(βX1lj)exp(βX1lj)+∑k=1mexp(βX2ljk).

The null hypothesis of no association *H*_0 _: *β *= 0 can be tested by the score statistic given by $Z_{\text{MTT}}(x)=U(x)/{\left\{{\hat{\text{Var}}}_{H_{0}}(U(x))\right\}}^{1/2}={\left(\partial \log L/\partial \beta \right)}_{H_{0}}/{\left\{-{\left(\partial^{2}\log L/\partial \beta^{2}\right)}_{H_{0}}\right\}}^{1/2}$. Using the matched case-control data, the closed form of the matching trend test (MTT) can be written as [21]

(3) $$\begin{array}{l} \;Z_{\text{MTT}}(x)=\frac{U(x)}{{\left[V(x)\right]}^{1/2}}\\ =\frac{\sum_{l=1}^{L}\sum_{j=1}^{r_{l}}\left(mX_{1lj}-\sum_{k=1}^{m}X_{2ljk}\right)}{{\left[\sum_{l=1}^{L}\sum_{j=1}^{r_{l}}\left\{(m+1)(X_{1lj}^{2}+\sum_{k=1}^{m}X_{2ljk}^{2})-{\left(X_{1lj}+\sum_{k=1}^{m}X_{2ljk}\right)}^{2}\right\}\right]}^{1/2}}. \end{array}$$

Obviously, $\sum_{j=1}^{r_{l}}X_{1lj}=r_{2l}+xr_{1l}$ and $\sum_{j=1}^{r_{l}}\sum_{k=1}^{m}X_{2ljk}=s_{2l}+xs_{1l}$. *Z*_MTT_(*x*), follows *N*(0,1) under the null hypothesis of no association.

Suppose a family of scientifically plausible models is defined. Similar to the CATTs, corresponding to each model, an asymptotically optimal normally distributed MTT can be obtained. For example, *Z*_MTT_(0), *Z*_MTT_(0.5) and *Z*_MTT_(1) are optimal for the REC, ADD and DOM models respectively. When the genetic model is uncertain, a pre-specified test from this family is not fully efficient, hence, MTTs are not suggested to be directly used when the underlying genetic model is unknown. This underlying genetic model, however, can be ascertained using the Hardy-Weinberg Disequilibrium (HWD) coefficient which is de-noted as Δ = *Pr*(*DD*) - [*Pr*(*DD*)+*Pr*(*Dd*)/2]^2^. In the unmatched study, denote the HWD coefficients in the case group and the control group as Δ*_p _*= *Pr*(*DD|case*) - [*Pr*(*DD|case*)+*Pr*(*Dd|case*)/2]^2 ^and Δ*_q _*= *Pr*(*DD|control*) - [*Pr*(*DD|control*) + *Pr*(*Dd|control*)/2]^2^, Zheng and Ng [11] obtained that Δ_*p *_- Δ_*q *_> 0 under REC and Δ_*p *_- Δ_*q *_< 0 under DOM. Using the matched design described above, we denote Δ*_pl _*and Δ*_ql _*as the HWD coefficients in the case group and the control group of the *l*th sub-population respectively, *l *= 1,..., *L*. Similar to the unmatched counterpart, we have Δ_*pl *_- Δ*_ql _*> 0 for each *l*, *l *= 1,..., *L *thus $\sum_{l=1}^{L}\left(\Delta_{pl}-\Delta_{ql}\right)>0$ under REC and Δ_*pl *_- Δ_*ql *_< 0 for each *l*, *l *= 1,..., *L *thus $\sum_{l=1}^{L}\left(\Delta_{pl}-\Delta_{ql}\right)<0$ under DOM.

Denote ${\hat{\Delta }}_{l}={\hat{\Delta }}_{pl}-{\hat{\Delta }}_{ql}=[{\hat{p}}_{2l}-{\left({\hat{p}}_{2l}+\frac{1}{2}{\hat{p}}_{1l}\right)}^{2}]-[{\hat{q}}_{2l}-{\left({\hat{q}}_{2l}+\frac{1}{2}{\hat{q}}_{1l}\right)}^{2}]$ where ${\hat{p}}_{il}=r_{il}/r_{l}$ and ${\hat{q}}_{il}=s_{il}/(mr_{l})$ for *i *= 0, 1, 2 and *l *= 1,..., *L*. Under the null hypothesis of no association and assume HWE in each stratum, using simple algebra we can obtain ${\hat{\lambda }}_{l}={\hat{Var}}_{H_{0}}({\hat{\Delta }}_{l})=(m+1){\left(1-{\hat{p}}_{l}\right)}^{2}{\hat{p}}_{l}{}^{2}/(r_{l}m)$ where ${\hat{p}}_{l}=[2(r_{2l}+s_{2l})+(r_{1l}+s_{1l})]/[2(m+1)r_{l}]$. Thus, using the same motivation as the Cochran-Mantel-Haenszel (CMH) statistic [1,25,26] we can construct the stratified model reduction test (SMRT):

(4) $$\begin{array}{c} Z_{\text{SMRT}}=\frac{\hat{\Delta }}{\sqrt{\hat{\lambda }}}=\frac{{\sum_{l=1}^{L}\hat{\Delta }}_{l}}{\sqrt{\sum_{l=1}^{L}{\hat{\lambda }}_{l}}}\\ =\frac{\sum_{l=1}^{L}\left\{[{\hat{p}}_{2l}-{\left({\hat{p}}_{2l}+\frac{1}{2}{\hat{p}}_{1l}\right)}^{2}]-[{\hat{q}}_{2l}-{\left({\hat{q}}_{2l}+\frac{1}{2}{\hat{q}}_{1l}\right)}^{2}]\right\}}{\sqrt{\sum_{l=1}^{L}\left(m+1){\left(1-{\hat{p}}_{l}\right)}^{2}\hat{p}\iota^{2}/(r_{l}m\right)}}. \end{array}$$

Notice that the denominator of *Z*_SMRT _is estimated under the null hypothesis thus *Z*_SMRT _is a score test [27]. We may also use the Wald test or likelihood ratio test. However, if we adopt the Wald test, the statistic becomes much more complex; if we adopt the likelihood ratio test, the statistic cannot be expressed explicitly thus it is hard to derive the correlations between the two stage tests and calculate the p-value of the overall test. For these reasons, the score test is adopted. Under the null hypothesis, *Z*_SMRT _asymptotically follows a standard normal distribution *N*(0, 1). *Z*_SMRT _tends to be large if the true genetic model is REC and tends to be small if the true genetic model is DOM. Hence, with a pre-specified threshold *c *> 0 (set to be Φ^-1^(0.95)), we can classify the underlying genetic model as REC if *Z*_SMRT _>*c*, DOM if *Z*_SMRT _< -*c *and ADD otherwise. So when the underlying genetic model is decided, *Z*_MTT_(*x*) optimal for the corresponding genetic model can be used to test for association. Notice that in the above discussion, we assume that D is the risk allele. If D is the risk allele as we assume, *Z*_MTT_(0) and *Z*_MTT_(1) are optimal for the REC and DOM models respectively. On the other hand, if d is the risk allele, then *Z*_MTT_(0) and *Z*_MTT_(1) are optimal for the DOM and REC models respectively. Besides, the expected values of *Z*_MTT_(0) and *Z*_MTT_(1) are negative in this case. Similar to Joo et al. [13], we use *Z*_MTT_(0.5) to determine the risk allele. That is, if *Z*_MTT_(0.5) > 0, *Z*_MTT_(0), *Z*_MTT_(0.5), *Z*_MTT_(1) are optimal for the REC, ADD and DOM models; if *Z*_MTT_(0.5) ≤ 0, -*Z*_MTT_(1), -*Z*_MTT_(0.5), -*Z*_MTT_(0) are optimal for the REC, ADD and DOM models. Hence, the stratified genetic model selection (SGMS) test is proposed as

(5) $$\begin{array}{ll} Z_{\text{SGMS}} & =\{\begin{array}{ll} Z_{\text{MTT}}(0)I(Z_{\text{MTT}}(0.5)>0)-Z_{\text{MTT}}(1)I(Z_{\text{MTT}}(0.5)\leq 0) & \text{if}\;Z_{\text{SMRT}}>c\\ \text{sign}(Z_{\text{MTT}}(0.5))Z_{\text{MTT}}(0.5) & \text{if}|Z_{\text{SMRT}}|\leq c\\ Z_{\text{MTT}}(1)I(Z_{\text{MTT}}(0.5)>0)-Z_{\text{MTT}}(0)I(Z_{\text{MTT}}(0.5)\leq 0) & \text{if}\;Z_{\text{SMRT}}<-c. \end{array} \end{array}$$

Under the null hypothesis of no association, we show that (*Z*_MTT_(0.5), *Z*_SMRT_, *Z*_MTT_(*x*)) asymptotically follows a multivariate normal distribution *N*(0, ∑*_x_*) where

$\Sigma_{x}=\left(\begin{array}{ccc} 1 & 0 & \rho_{x,0.5}\\ 0 & 1 & \rho_{x}\\ \rho_{x,0.5} & \rho_{x} & 1 \end{array}\right),$

*x *= 0, 1. In addition, *Z*_MTT_(0.5) and *Z*_SMRT _are asymptotically independent. Detailed proof and the forms of *ρ_x _*and *ρ_x_*,_0.5 _as well as their consistent estimates are derived in the **Appendix**. Define **ø***_x_*(*z*_1_, *z*_2_, *z*_3_) as the density function of *N*(0, **∑***_x_*) and *ø*(*z*) as the density function of the standard normal distribution. Let *t *> 0 be the observed value of *Z*_SGMS _and the corresponding p-value is obtained as

(6) $$\begin{array}{c} PV_{s}=Pr(Z_{\text{SGMS}}>t)\\ =2\left\{\sum_{x=0,1}\int_{t}^{+\infty }\int_{c}^{+\infty }\int_{0}^{+\infty }\phi_{x}(z_{1},z_{2},z_{3})dz_{1}dz_{2}dz_{3}\right\}\\ +2\left\{\int_{-c}^{c}\phi (z)dz\int_{t}^{+\infty }\phi (z)dz\right\}. \end{array}$$

With a pre-specified significance level *ζ*, we declare a significant association if *PV_s _*<*ζ*.

Although Z_SMRT _can be used to determine the underlying genetic model, the probability of selecting the correct genetic model is low when the GRRs are small or moderate. On the other hand, the probability of correctly excluding the most unlikely genetic model remains high when GRRs are very small. That is, when *Z*_SMRT _>*c*, the underlying genetic model is likely to be either REC or ADD rather than just REC and excluding the DOM model is more reasonable than just selecting the REC model. Similarly, when *Z*_SMRT _< -*c*, excluding REC is more reasonable than just selecting the DOM model. Therefore, when the GRRs are low, the strategy of excluding the most unlikely genetic model is more preferred to that of selecting the most suitable genetic model.

Similar to Joo et al. [13], we define the MERT-type statistic named the matching averaged test (MAT) as $Z_{\text{MAT}}(0)=(Z_{\text{MTT}}(0)+Z_{\text{MTT}}(0.5))/\sqrt{2(1+{\hat{\rho }}_{0,05})}$ and $Z_{\text{MAT}}(1)=(Z_{\text{MTT}}(1)+Z_{\text{MTT}}(0.5))/\sqrt{2(1+{\hat{\rho }}_{1,0.5})}$. The definition of MATs indicate that *Z*_MAT_(0) is optimal for either REC or ADD and *Z*_MAT_(1) is optimal for either DOM or ADD. Besides, Z_MTT_(0.5) is still optimal for just ADD. Utilizing the stratified genetic model exclusion (SGME) strategy, we use *Z*_MAT_(0) to test for association if *Z*_SMRT _>*c *thus DOM is excluded; use *Z*_MAT_(1) if *Z*_SMRT _< -*c *thus REC is excluded and *Z*_MTT_(0.5) otherwise. In addition, similar to SGMS, *Z*_MTT_(0.5) is used at the beginning to determine the risk allele. Hence, the statistic for the SGME approach can be written as

(7) $$Z_{\text{SGME}}=\{\begin{array}{cc} Z_{\text{MAT}}(0)I(Z_{\text{MTT}}(0.5)>0)-Z_{\text{MAT}}(1)I(Z_{\text{MTT}}(0.5)\leq 0) & \text{if }Z_{\text{SMRT}}>c\\ \begin{array}{l} \text{sign}(Z_{\text{MTT}}(0.5))Z_{\text{MTT}}(0.5)\\ Z_{\text{MAT}}(1)I(Z_{\text{MTT}}(0.5)>0)-Z_{\text{MAT}}(0)I(Z_{\text{MTT}}(0.5)\leq 0) \end{array} & \begin{array}{l} \text{if}|Z_{\text{SMRT}}|\leq c\\ \text{if }Z_{\text{SMRT}}<-c \end{array} \end{array}$$

Under the null hypothesis of no association, we obtain that (*Z*_MTT_(0.5), *Z*_SMRT_, *Z*_MAT_(*x*)) asymptotically follows a multivariate normal distribution $N(0,\;\Sigma_{x}^{*})$ where

$$\Sigma_{x}^{*}=\left(\begin{array}{ccc} 1 & 0 & \sqrt{\frac{\left(1+\rho_{x,0.5}\right)}{2}}\\ 0 & 1 & \frac{\rho_{x}}{\sqrt{2(1+\rho_{x,0.5})}}\\ \sqrt{\frac{\left(1+\rho_{x,0.5}\right)}{2}} & \frac{\rho_{x}}{\sqrt{2(1+\rho_{x,0.5})}} & 1 \end{array}\right),$$

*x*= 0, 1. Define $\phi_{x}^{*}(z_{1},\;z_{2},\;z_{3})$ as the density function of $N(\text{O},\;\Sigma_{x}^{*})$, similar to the test of *Z*_SGMS_, the p-value of *Z*_SGME _can be derived as

(8) $$\begin{array}{c} PV_{e}=Pr(Z_{\text{SGME}}>t)\\ =2\left\{\sum_{x=0,1}\int_{t}^{+\infty }\int_{c}^{+\infty }\int_{0}^{+\infty }\phi_{x}^{*}(z_{1},z_{2},z_{3})dz_{1}dz_{2}dz_{3}\right\}\\ +2\left\{\int_{-c}^{c}\phi (z)dz\int_{t}^{+\infty }\phi (z)dz\right\}. \end{array}$$

We declare a significant association if *PV_e _*<*ζ *where *ζ *is the pre-specified significance level.

### Other robust procedures

In equation (2), we use one indicator to code three genotypes. One the other hand, if we define two dummy variables ((*X*_1*lj*1_, *X*_1*lj*2_) for the cases and (*X*_2*ljk*1_, *X*_2*ljk*2_) for the controls) taking values (0,0), (0,1) and (1,1) to code three genotypes *G*_0_, *G*_1 _and *G*_2_, the conditional likelihood function becomes [10]

(9) $$\begin{array}{l} \;L(\beta_{1},\beta 2|G,C_{1},...,C_{L})\\ =\;\prod_{l=1}^{L}\prod_{j=1}^{r_{l}}\frac{\exp(\beta_{1}X_{1lj1}+\beta_{2}X_{1lj2})}{\exp(\beta_{1}X_{1lj1}+\beta_{2}X_{1lj2})+\sum_{k=1}^{m}\exp(\beta_{1}X_{2ljk1}+\beta_{2}X_{2ljk2})}. \end{array}$$

The score test derived from equation (9), denoted by $Z_{\chi^{2}}$ with 2 df, has an asymptotic *χ*^2 ^distribution with 2 df under *H*_0 _: *β*_1 _= *β*_2 _= 0. Note that $Z_{\chi^{2}}$ with 2 df does not rely on any information of the underlying genetic model so it is a robust test against model of inheritance.

Another robust test is the MAX3 which was also proposed as an efficiency robust test for unmatched genetic association studies [7,28]. Analogy to the unmatched counterpart, Zheng and Tian [21] proposed the MAX3 statistic for matched case-control association study which is defined as

$$Z_{\text{MAX}3}=max(|Z_{\text{MTT}}(0)|,|Z_{\text{MTT}}(0.5)|,|Z_{\text{MTT}}(1)|).$$

Compared with the optimal MTTs and *χ*^2 ^test with 2 df, MAX3 has the largest minimum power across the three genetic models [21,29]. As mentioned in Zheng and Tian [21] and Joo et al. [10], the null distribution of *Z*_MAX3 _can be approximated by Monte-Carlo simulation. In addition, the p-value of *Z*_MAX3 _can also be obtained according to the asymptotic formula given by Zang et al. [29].

## Results

### Simulation

To check whether GMS and GME can keep the correct size in the presence of confounding factors, we carried out simulation studies to examine the performance of GMS and GME in the presence of sub-populations. The nominal level was set at 0.05. We assumed that due to confounding factors, each of the case and control populations was divided into two sub-populations with equal probability. The simulation results are summarized in Table 1. We find that when there are no confounding factors (Scenario *1 *), GMS and GME can control the size well. On the other hand, in the presence of confounding factors and adopt the matched design to each of the sub-populations, GMS and GME are found to be conservative (Scenarios *2*, *3 *and *4 *). Furthermore, without the matched design, the type I error rates of GMS and GME are seriously inflated (Scenarios *5 *to *8 *). The simulation results show that in the presence of sub-populations, GMS and GME cannot keep the correct size whether or not the matched design is utilized.

**Table 1:** Type I error rates of GMS and GME based on 10,000 replicates without confounding (Scenario 1) and in the presence of confounding factors (Scenarios 2-8), with the significance level 0.05 using *r_l _*cases and *s_l _*controls; *p_l _*is the risk allele frequency and *k_l _*is the prevalence, *l *= 1,2.

Scenario	***r***_**1**_	***r***_**2**_	***s***_**1**_	***s***_**2**_	***p***_**1**_	***p***_**2**_	***k***_**1**_	***k***_**2**_	GMS	GME
*1*	250	250	250	250	0.3	0.3	0.05	0.05	0.0510	0.0502
										
*2*	250	250	250	250	0.05	0.5	0.01	0.1	0.0199	0.0141
*3*	250	250	250	250	0.1	0.5	0.01	0.1	0.0190	0.0167
*4*	250	250	250	250	0.2	0.4	0.03	0.07	0.0391	0.0384
										
*5*	300	200	200	300	0.2	0.4	0.03	0.07	0.3923	0.4403
*6*	325	175	175	325	0.2	0.4	0.03	0.07	0.7337	0.7880
*7*	350	150	150	350	0.2	0.4	0.03	0.07	0.9077	0.9567
*8*	375	125	125	375	0.2	0.4	0.03	0.07	0.9625	0.9954

To check if the ability of *Z*_SMRT _to select the correct genetic model is low when GRRs are small, we conducted a simulation to compare the selection procedure with the exclusion procedure. Considered 300 cases with 600 matched controls, the samples were divided into 3 sub-populations with proportions being 0.3, 0.3 and 0.4 respectively. Set the MAFs and the penetrance in the three strata as (*p*_1_, *p*_2_, *p*_3_) = (0.1, 0.3, 0.5) and (*f*_01_, *f*_02_, *f*_03_) = (0.01, 0.05, 0.02). The threshold *c *was fixed as Φ^-1^(0.95) and let GRR2 = *λ*_2*l *_increase from 1.1 to 2.0 with increments of 0.1, *l *= 1,..., *L*.

**Figure 1:**
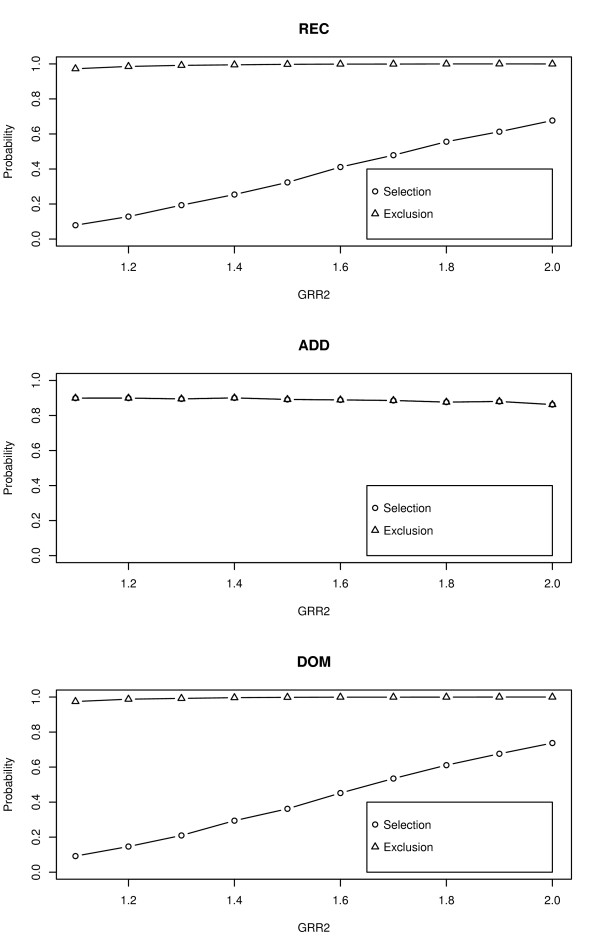
**The probabilities of correctly selecting the genetic models and of correctly excluding the most unlikely genetic models based on 10,000 replicates**.

The results are summarized in Figure 1, with circles representing the probabilities of selecting the correct genetic models and triangles representing the probabilities of correctly excluding the most unlikely genetic models. From Figure 1 we can find that under REC and DOM the triangles are always higher than 90%, whereas the circles can be less than 20% when the GRR2 is small. However, when ADD is the true genetic model, the circles coincide with the triangles. This is because under ADD, both REC and DOM are the most unlikely models thus the selection procedure is just the same as the exclusion procedure.

Next, we performed simulations with no disease association and under various genetic models to evaluate the performance of the proposed robust methods. Moreover, we also considered the MTTs optimal for the REC, ADD and DOM models, i.e. *Z*_MTT_(0), *Z*_MTT_(0.5) and *Z*_MTT_(1) respectively. Let *R*, *S*, *F_i_*, *K *and *P *denoted the vectors of *r_l_*, *s_l_*, *f_il_*, *k_l _*and *p_l _*respectively across sub-populations, *l *= 1,..., *L*, *i *= 0, 1, 2. Each sub-population was in HWE. We first examined the type I error rates of the mentioned tests under the null hypothesis of no association with nominal levels taken as 0.05 and 0.01 respectively. The results are summarized in Table 2.

**Table 2:** Type I error rates of *Z*_MTT(0)_, *Z*_MTT(0.5)_, *Z*_MTT(1)_, *Z*_SGMS_, *Z*_SGME_, *Z*_MAX3 _and $Z_{\chi^{2}}$ with 2 df based on 10,000 replicates in the presence of confounding factors with the significance level *α *using *R *cases and *S *controls.

Scenario	*α*	*Z*_MTT_(0)	*Z*_MTT_(0.5)	*Z*_MTT_(1)	*Z*_SGMS_	*Z*_SGME_	*Z*_MAX3_	$Z_{\chi^{2}}$
*A*	0.05	0.0527	0.0498	0.0518	0.0501	0.0490	0.0527	0.0531
*B*		0.0487	0.0503	0.0493	0.0494	0.0502	0.0515	0.0481
*C*		0.0510	0.0512	0.0509	0.0506	0.0510	0.0513	0.0490
*D*		0.0526	0.0524	0.0537	0.0529	0.0528	0.0516	0.0484
*E*		0.0519	0.0512	0.0534	0.0536	0.0526	0.0501	0.0507
*F*		0.0485	0.0486	0.0479	0.0488	0.0467	0.0501	0.0481
*G*		0.0493	0.0497	0.0490	0.0492	0.0498	0.0457	0.0491
*H*		0.0522	0.0493	0.0480	0.0522	0.0521	0.0525	0.0522
								
*A*	0.01	0.0092	0.0081	0.0100	0.0083	0.0081	0.0075	0.0084
*B*		0.0096	0.0091	0.0106	0.0106	0.0103	0.0096	0.0101
*C*		0.0093	0.0101	0.0109	0.0108	0.0104	0.0101	0.0109
*D*		0.0121	0.0101	0.0098	0.0093	0.0093	0.0095	0.0105
*E*		0.0098	0.0094	0.0101	0.0093	0.0092	0.0083	0.0114
*F*		0.0109	0.0095	0.0106	0.0109	0.0102	0.0102	0.0109
*G*		0.0100	0.0094	0.0105	0.0114	0.0103	0.0111	0.0093
*H*		0.0087	0.0111	0.0104	0.0099	0.0103	0.0117	0.0107

*A *: *R *= (150, 150, 200), *S *= (300, 300, 400), *P *= (0.1, 0.3, 0.5), *K *= (0.01, 0.05, 0.02)

*B *: *R *= (100, 300, 100), *S *= (200, 600, 200), *P *= (0.1, 0.3, 0.5), *K *= (0.01, 0.05, 0.02)

*C *: *R *= (300, 300, 400), *S *= (300, 300, 400), *P *= (0.1, 0.3, 0.5), *K *= (0.01, 0.05, 0.02)

*D *: *R *= (200, 600, 200), *S *= (200, 600, 200), *P *= (0.1, 0.3, 0.5), *K *= (0.01, 0.05, 0.02)

*E *: *R *= (250, 250), *S *= (500, 500), *P *= (0.2, 0.4), *K *= (0.01, 0.02)

*F *: *R *= (150, 350), *S *= (300, 700), *P *= (0.2, 0.4), *K *= (0.01, 0.02)

*G *: *R *= (500, 500), *S *= (500, 500), *P *= (0.2, 0.4), *K *= (0.01, 0.02)

*H *: *R *= (300, 700), *S *= (300, 700), *P *= (0.2, 0.4), *K *= (0.01, 0.02)

We considered eight separate scenarios (A to H) with different numbers of cases, controls, risk allele frequencies and disease prevalences. For example, in scenario A, 150, 150 and 200 cases from 3 different sub-populations comprised the whole case group and each case was matched with 2 controls within the same sub-population. The risk allele frequencies of the 3 sub-populations were 0.1, 0.3 and 0.5 respectively and the disease prevalences equalled to 0.01, 0.05 and 0.02. Table 2 shows that the type I error rates of all the mentioned tests are close to the nominal levels and so the robust tests and MTTs can control the sizes well. Besides, although we assume that HWE holds in each sub-population, a moderate departure from HWE has little impact to the sizes of SGMS and SGME (results skipped for brevity).

We also conducted simulation to investigate the performance of the proposed tests for small sized samples, where the number of cases is at most 100. The results are summarized in Table 3. The settings were the same as those in Table 2 except that the sample sizes in Table 3 were only 10% of those in Table 2. The results show that the proposed tests can keep the size reasonably well even for small sample case.

**Table 3:** Type I error rates of *Z*_MTT(0)_, *Z*_MTT(0.5)_, *Z*_MTT(1)_, *Z*_SGMS_, *Z*_SGME_, *Z*_MAX3 _and $Z_{\chi^{2}}$ with 2 df for small sample size.

Scenario	*α*	*Z*_MTT_(0)	*Z*_MTT_(0.5)	*Z*_MTT_(1)	*Z*_SGMS_	*Z*_SGME_	*Z*_MAX3_	$Z_{\chi^{2}}$
A*	0.05	0.0474	0.0501	0.0487	0.0493	0.0495	0.0452	0.0502
B*		0.0531	0.0479	0.0497	0.0480	0.0485	0.0462	0.0503
C*		0.0569	0.0526	0.0489	0.0507	0.0526	0.0516	0.0488
D*		0.0470	0.0480	0.0516	0.0482	0.0484	0.0488	0.0503
E*		0.0492	0.0498	0.0489	0.0518	0.0511	0.0535	0.0498
F*		0.0519	0.0503	0.0514	0.0535	0.0537	0.0486	0.0489
G*		0.0484	0.0505	0.0526	0.0483	0.0466	0.0551	0.0502
H*		0.0504	0.0453	0.0451	0.0451	0.0456	0.0484	0.0504
								
A*	0.01	0.0076	0.0083	0.0092	0.0102	0.0089	0.0108	0.0126
B*		0.0075	0.0091	0.0097	0.0078	0.0088	0.0086	0.0081
C*		0.0078	0.0089	0.0096	0.0082	0.0084	0.0126	0.0092
D*		0.0080	0.0095	0.0116	0.0093	0.0092	0.0111	0.0099
E*		0.0072	0.0093	0.0098	0.0099	0.0092	0.0091	0.0120
F*		0.0087	0.0081	0.0089	0.0085	0.0081	0.0091	0.0116
G*		0.0077	0.0120	0.0120	0.0113	0.0125	0.0081	0.0102
H*		0.0079	0.0087	0.0088	0.0073	0.0081	0.0098	0.0095

The results are simulated based on 10,000 replicates in the presence of confounding factors with the significance level *α *using *R *cases and *S *controls.

A*: *R *= (15, 15, 20), *S *= (30, 30, 40), *P *= (0.1, 0.3, 0.5), *K *= (0.01, 0.05, 0.02)

B*: *R *= (10, 30, 10), *S *= (20, 60, 20), *P *= (0.1, 0.3, 0.5), *K *= (0.01, 0.05, 0.02)

C*: *R *= (30, 30, 40), *S *= (30, 30, 40), *P *= (0.1, 0.3, 0.5), *K *= (0.01, 0.05, 0.02)

D*: *R *= (20, 60, 20), *S *= (20, 60, 20), *P *= (0.1, 0.3, 0.5), *K *= (0.01, 0.05, 0.02)

E*: *R *= (25, 25), *S *= (50, 50), *P *= (0.2, 0.4), *K *= (0.01, 0.02)

F*: *R *= (15, 35), *S *= (30, 70), *P *= (0.2, 0.4), *K *= (0.01, 0.02)

G*: *R *= (50, 50), *S *= (50, 50), *P *= (0.2, 0.4), *K *= (0.01, 0.02)

H*: *R *= (30, 70), *S *= (30, 70), *P *= (0.2, 0.4), *K *= (0.01, 0.02)

The powers of the MTTs and robust tests were compared under three genetic models (REC, ADD and DOM). The settings were the same as those in Table 2 except that the nominal level was set to be 0.05 and the GRR was determined so that the optimal MTT has the maximum power of about 80%. The results are summarized in Table 4. In each row, the power of the robust test which performs best among the four robust tests considered in Table 4 is bold-faced.

**Table 4:** Empirical powers of *Z*_MTT(0)_, *Z*_MTT(0.5)_, *Z*_MTT(1)_, *Z*_SGMS_, *Z*_SGME_, *Z*_MAX3 _and $Z_{\chi^{2}}$ with 2 df based on 10,000 replicates.

Scenario	Model	**Z**_**MTT**_**(0)**	**Z**_**MTT**_**(0.5)**	**Z**_**MTT**_**(1)**	*Z*_SGMS_	*Z*_SGME_	*Z*_MAX3_	$Z_{\chi^{2}}$	ρ*
*A*	REC	0.8059	0.5590	0.1369	0.6981	0.6760	**0.7340**	0.7154	0.3267
	ADD	0.4890	0.7998	0.7126	0.7594	**0.7896**	0.7629	0.7188	0.7623
	DOM	0.1237	0.6818	0.8040	0.7142	0.7155	**0.7300**	0.7158	0.2639
*B*	REC	0.8073	0.5367	0.1356	0.6725	0.6497	**0.7229**	0.7147	0.3423
	ADD	0.4637	0.7977	0.7258	0.7646	**0.7908**	0.7502	0.7140	0.7445
	DOM	0.1287	0.7011	0.8054	0.7168	0.7259	**0.7383**	0.7199	0.2756
*C*	REC	0.8057	0.5503	0.1330	0.6970	0.6691	**0.7244**	0.7153	0.3038
	ADD	0.4896	0.8052	0.7139	0.7654	**0.7952**	0.7648	0.7094	0.7295
	DOM	0.1193	0.6877	0.8062	0.7153	0.7210	**0.7445**	0.7112	0.2710
*D*	REC	0.7978	0.5235	0.1400	0.6655	0.6433	**0.7177**	0.7144	0.3124
	ADD	0.4639	0.8045	0.7308	0.7654	**0.7934**	0.7499	0.7090	0.7037
	DOM	0.1204	0.7024	0.8071	0.7144	0.7225	**0.7250**	0.7033	0.2792
*E*	REC	0.7974	0.5276	0.1453	0.7166	0.6782	**0.7288**	0.7218	0.3492
	ADD	0.4667	0.8014	0.7294	0.7554	**0.7884**	0.7517	0.7131	0.7396
	DOM	0.1261	0.6989	0.8027	0.7135	**0.7234**	0.7161	0.7077	0.2795
*F*	REC	0.8056	0.5547	0.1535	0.6991	0.6742	**0.7317**	0.7173	0.3561
	ADD	0.5014	0.8068	0.7195	0.7650	**0.7955**	0.7524	0.7112	0.7661
	DOM	0.1389	0.6933	0.8003	0.7167	0.7231	**0.7291**	0.7141	0.2887
*G*	REC	0.8045	0.5241	0.1499	0.7233	0.6786	**0.7316**	0.7082	0.3172
	ADD	0.4562	0.8020	0.7313	0.7522	**0.7883**	0.7560	0.7068	0.6970
	DOM	0.1247	0.7091	0.8004	0.7167	0.7297	**0.7468**	0.7099	0.2825
*H*	REC	0.7967	0.5481	0.1467	0.6950	0.6651	**0.7203**	0.7136	0.3279
	ADD	0.4885	0.8017	0.7154	0.7610	**0.7911**	0.7529	0.7009	0.7272
	DOM	0.1254	0.6944	0.8055	0.7183	0.7248	**0.7366**	0.7091	0.2945

The settings are the same as those in Table 2 except that the GRRs are determined so that the optimal MTT has the maximum power of about 80%. The significance level is 0.05. ρ* is the minimum correlation of the optimal tests.

From Table 4 we notice that although the MTTs can obtain the highest power if the genetic models are correctly specified, the minimum powers of *Z*_MTT_(0) and *Z*_MTT_(1) are below 20% and the minimum powers of *Z*_MTT_(0.5) are between 50% to 60%. On the other hand, the minimum powers of the robust tests are about 65% across all genetic models. Table 4 clearly shows the advantage of the robust tests that, when the genetic model is unknown, the robust tests are more preferred than the MTTs. Besides, from Table 4 we can conclude that if only the REC, ADD and DOM models are considered, *Z*_SGME _and *Z*_MAX3 _perform better than the other two robust tests and *Z*_MAX3 _always dominate $Z_{\chi^{2}}$ with 2 df under such situations. Table 4 also reports ρ*, which is defined as the minimum correlation of the optimal tests [5]. For example, when REC is the true model, then ρ* = min(corr(*Z*_MTT_(0), *Z*_MTT_(0.5)), corr(*Z*_MTT_(0), *Z*_MTT_(1))). ρ* is considered here as a guideline for choosing efficiency robust tests between *Z*_SGME _and *Z*_MAX3_. From Table 4 we find that when ρ* is small (around 0.3), *Z*_MAX3 _performs better or at least as powerful as *Z*_SGME_. However, when ρ* is large (around 0.7), Z_SGME _is a better choice. Notice that this finding is similar to the property of the efficiency robust procedures in survival data analysis studied by Freidlin et al. [30] who also suggest to use the MAX-type statistic if ρ* is less than 0.6 or 0.7.

**Figure 2:**
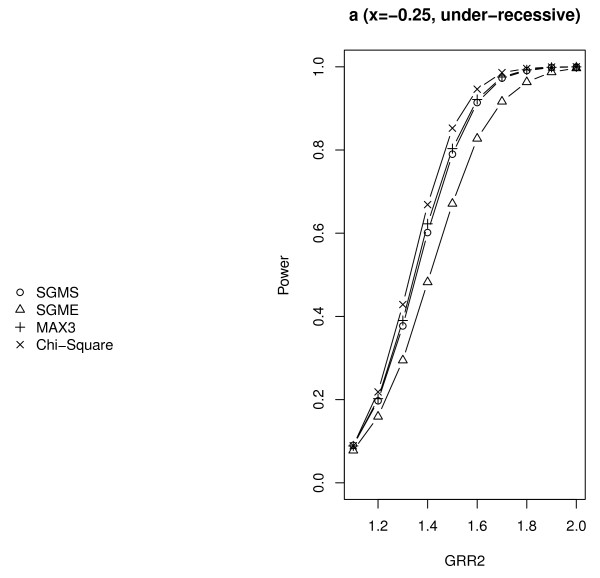
**Empirical powers of *Z*_SGMS_, *Z*_SGME_, *Z*_MAX3 _and $Z_{\chi^{2}}$ with 2 df under genetic model a**. The significance level is 0.05.

**Figure 3:**
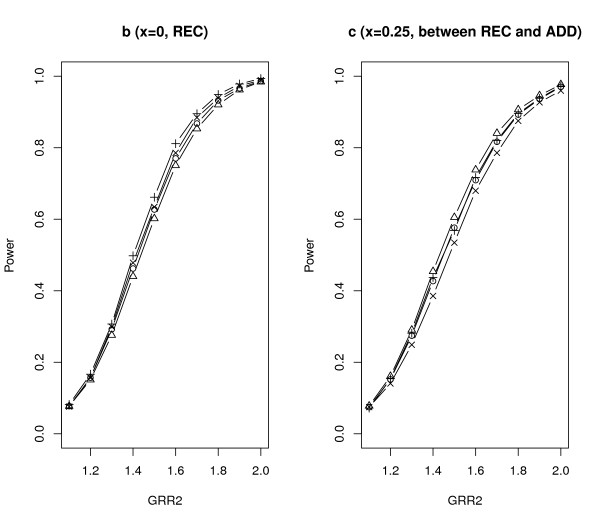
**Empirical powers of *Z*_SGMS_, *Z*_SGME_, *Z*_MAX3 _and $Z_{\chi^{2}}$ with 2 df under genetic models b and c**. The significance level is 0.05.

**Figure 4:**
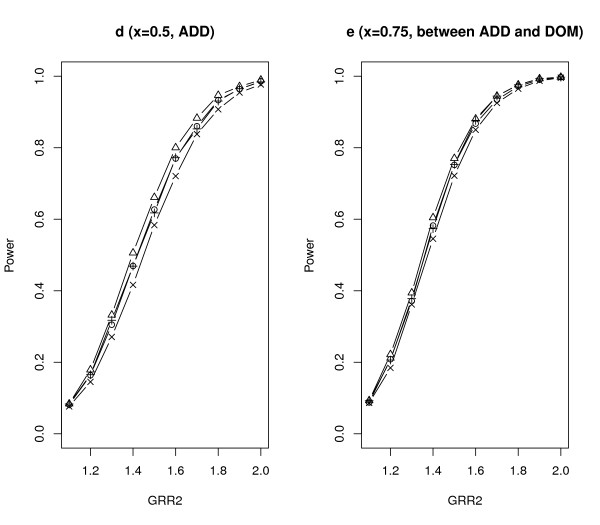
**Empirical powers of *Z*_SGMS_, *Z*_SGME_, *Z*_MAX3 _and $Z_{\chi^{2}}$ with 2 df under genetic models d and e**. The significance level is 0.05.

**Figure 5:**
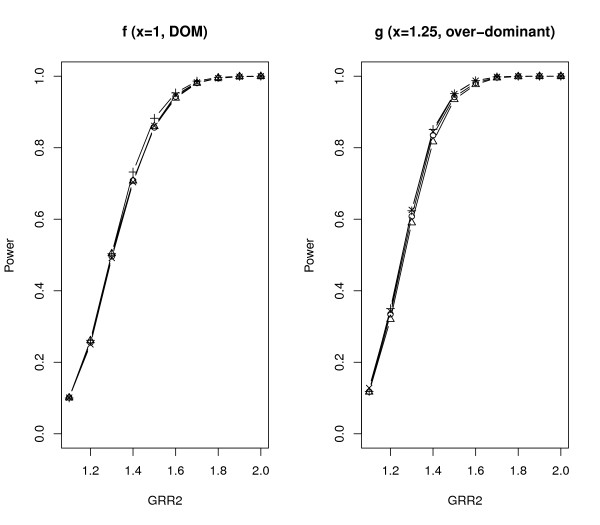
**Empirical powers of *Z*_SGMS_, *Z*_SGME_, *Z*_MAX3 _and $Z_{\chi^{2}}$ with 2 df under genetic models f and g**. The significance level is 0.05.

We further compared *Z*_SGMS_, *Z*_SGME_, *Z*_MAX3 _and $Z_{\chi^{2}}$ with 2 df under different genetic models. The parameter settings were the same as those of scenario *A *in Table 2 except that *λ*_2 _increased from 1.1 to 2.0 with increments of 0.1 and *λ*_1*l *_= 1 + *x*(*λ*_2*l *_- 1). The results are summarized in Figures 2, 3, 4 and 5 with titles *a*, *b*, *c*, *d*, *e*, *f*, *g *representing *x *= 0.25, 0, 0.25, 0.5, 0.75, 1, 1.25 respectively.

Notice that *x *= 0, 0.5, 1 (figures b, d, f) correspond to the REC, ADD and DOM models respectively. Under these three commonly used genetic models, *Z*_SGMS_, *Z*_SGME _and *Z*_MAX3 _have comparable powers although *Z*_MAX3 _may be slightly more powerful than the other two tests under the REC and DOM models, and *Z*_SGME _may dominate *Z*_MAX3 _and *Z*_SGMS _under the ADD model. $Z_{\chi^{2}}$ with 2 df has the least power among all the tests considered here. *x *= 0.25 (figure c) indicates a genetic model between REC and ADD and *x *= 0.75 (figure e) corresponds to a genetic model between ADD and DOM. The performance of the robust tests under such two genetic models is similar to that under ADD. *Z*_SGME _is slightly more powerful than *Z*_SGMS _and *Z*_MAX3_, and $Z_{\chi^{2}}$ with 2 df still obtains the least power.

*x *= - 0.25 and 1.25 indicate two less plausible models, the under-recessive model (figure a) and over-dominant model (figure g). Under the under-recessive model where *f*_1*l *_<*f*_0*l*_, $Z_{\chi^{2}}$ with 2 df is the most powerful test followed by *Z*_SGMS _and *Z*_MAX3_. *Z*_SGME _performs the worst in such a situation. Under the over-dominant model where *f*_1*l *_>*f*_2*l*_, all the robust tests perform very similarly.

To summarize, if the mean genetic effect of the heterozygous genotype is between those of the two homozygous genotypes, then we suggest *Z*_MAX3_, *Z*_SGMS _and *Z*_SGME_. On the other hand, if the genetic effects are not ranked in accordance with the genotypes, then $Z_{\chi^{2}}$ with 2 df is preferred. This is reasonable because $Z_{\chi^{2}}$ with 2 df does not take the order of the genetic effects into consideration so it should perform well if the genetic effects are not ranked in accordance with the genotypes.

Notice that in our simulation we consider the common disease common variant (CDCV) which is currently the most popular theory underlying complex disease etiology. However, if the common disease rare variant (CDRV) assumption holds which implies that the disease etiology is caused collectively by multiple rare variants with moderate to high penetrances, the proposed tests perform conservatively and underpowered for detecting association [31]. In this case, the combined multivariate and collapsing (CMC) method proposed by Li and Leal [31] may be used to increase the power of the proposed tests.

### An application

We applied MTTs and the robust tests to a matched pair case-control etiologic study of sarcoidosis (ACCESS) [15]. In this study, a total of 497 matched pairs of case-control sets samples based on their age (within 5 years), race (Caucasian and African-American) and gender were recruited to test for association between immunoglobulin gene polymorphism and sarcoidosis. A subset containing 219 African-American matched pairs was used by Zheng and Tian [21]. We consider the KM(1,3) polymorphism as the candidate marker. After estimating the risk allele frequencies in controls of the matched sets defined by the two confounding factors (gender and race), we find three sub-populations namely Caucasian, Female African-American and Male African-American. The details of the matched data and sub-structure information are summarized in Table 5.

**Table 5:** The pair-matched case-control study of ACCESS.

		Controls			
			
Caucasian		'11'	'13'	'33'	Total
Cases	'11'	0	0	1	1
	'13'	0	9	36	45
	'33'	2	29	201	232

	Total	2	38	238	278
		Controls			
			
Female/African-American		'11'	'13'	'33'	Total

Cases	'11'	1	11	8	20
	'13'	8	26	40	74
	'33'	4	34	24	62

	Total	13	71	72	156
		Controls			
			
Male/African-American		'11'	'13'	'33'	Total

Cases	'11'	1	2	5	8
	'13'	1	14	17	32
	'33'	1	11	11	23

	Total	3	27	33	63
		Controls			
			
Combined		'11'	'13'	'33'	Total

Cases	'11'	2	13	14	29
	'13'	9	49	93	151
	'33'	7	74	236	317

	Total	18	136	343	497

First we applied the MTTs optimal for the REC, ADD and DOM models to the data set and obtained the p-values being 0.058, 0.025 and 0.093 for *Z*_MTT(0)_, *Z*_MTT(0.5) _and *Z*_MTT(1) _respectively. Thus, whether or not there is a significant association is unclear under a nominal level 0.05 because different genetic models give different answers.

Then we applied $Z_{\chi^{2}}$ with 2 df and *Z*_MAX3 _to the data set and obtained the p-values as 0.076 and 0.056, which were also hard to provide a more conclusive finding under a significance level of 0.05. Note that the p-value of *Z*_MAX3 _was calculated according to the asymptotic formula obtained by Zang et al. [29]. Thereafter we applied Z_SGMS _and Z_SGME _to the same data. We obtained *Z*_SMRT _= 0.124, which falls in the interval [-1.645,1.645] and strongly suggested an ADD model. Thus, for SGMS we select ADD and for SGME we exclude REC and DOM. Using formulas (6) and (8) we obtained the p-values as 0.0398 for *Z*_SGMS _and 0.0310 for *Z*_SGME_, both suggesting a marginally significant association. According to our simulation, *Z*_SGME _is the most powerful robust test under the ADD model. We also obtained the minimum correlation of the optimal tests ρ* = 0.603, which indicates that *Z*_SGME _is a better choice than *Z*_MAX3 _according to our previous discussion for Table 4. Obviously, our results are consistent with the findings in that discussion. To sum up, we observe that there is some association between the candidate marker and sarcoidosis.

## Discussion

In this paper, we extended the GMS [11] and GME [13] methods to the matched case-control association study and proposed the SGMS and SGME methods so that they can be used when there are confounding factors in the recruited samples. We showed that the p-values of both tests can be determined analytically based on the asymptotic tri-variate normal distributions. Besides, we also reviewed some other robust tests in matched case-control association study such as the MAX3 test and the *χ*^2 ^with 2 df test. Simulations were carried out to examine the robustness of all these tests. The tests were also used to analyze a real pair matched data set of sarcoidosis. Simulation results indicate that when the genetic model is unknown, a mis-specification of the genetic model may result in a substantial loss of power for the MTTs. In this situation, robust tests are preferred. Further comparisons among the robust tests were also conducted. According to our simulation, when the genetic effects are ordered in accordance with their genotypes, MAX3, SGMS and SGME are preferred. On the other hand, if the less plausible genetic models such as the over-dominant and under-recessive models cannot be excluded, then *χ*^2 ^with 2 df test is a good choice.

We adopted the matching framework in the stage of recruiting samples so our study is a pre-matched case-control association study. In practice, even in the unmatched case-control design matching is still an important tool to eliminate the effect of latent confounding factors such as the population stratification and cryptical relatedness. For example, Guan et al. [24] recently proposed a matched design in an unmatched case-control study. They post-matched individuals by their genotypes followed by a conditional matching analysis to correct for population stratification in genome-wide association studies. In fact, after applying their method or the principal components method [32] and its extension [33] to classify the latent population structure, all the robust tests discussed in this paper can be used as robust approaches as well as correcting the latent population stratification in the unmatched case-control or genome-wide association studies. The regression approach is also suggested in the literature to adjust for confounding factors other than markers. However, if the whole population has many subpopulation due to confounding, the performance of the regression method could be affected because too many nuisance parameters need to be estimated. Furthermore, how to derive the variance-covariance matrices of the distribution of the robust tests in this case is still uncertain. Further research in this area is needed.

## Conclusion

Simulation results and real data analysis show that SGMS and SGME can keep a correct Type I error rate for stratified data while have good efficiency robustness against genetic model uncertainty. Besides, the proposed formulas in this paper can easily be used to calculate the corresponding p-values. Thus, SGMS and SGME are useful for genetic data analysis of matched case-control design.

## Authors' contributions

ZY carried out the project and wrote the draft of the manuscript. FWK proposed the idea and revised the manuscript. Both authors read and approved the manuscript.

## Appendix

First we derive the correlation *ρ_x _*between *Z*_SMRT _and *Z*_MTT_(*x*). Define *U*(*x*) as the numerator of *Z*_MTT_, under the null hypothesis,

$$cov_{H_{0}}(\sum_{l=1}^{L}{\hat{\Delta }}_{l},U(x))=\sum_{l=1}^{L}cov_{H_{0}}({\hat{\Delta }}_{l},U(x))$$

Following Zheng and Ng [11], we can obtain that

$$\begin{array}{c} cov_{H_{0}}(\hat{△},U(0))=(m+1)\sum_{l=1}^{L}\left[p_{l}^{2}{\left(1-p_{l}\right)}^{2}\right]+O(1/r)\\ cov_{H_{0}}(\hat{△},U(0.5))=O(1/r)\\ cov_{H_{0}}(\hat{△},U(1))\;=-(m+1)\sum_{l=1}^{L}\left[p_{l}^{2}{\left(1-p_{l}\right)}^{2}\right]+O(1/r). \end{array}$$

When *n *→ ∞, $V(x)\to m(m+1)\sum_{l=1}^{L}r_{l}(x^{2}p_{1l}+p_{2l}-x^{2}p_{1l}^{2}-p_{2l}^{2}-2xp_{1l}p_{2l})$ and $\hat{\lambda }\to \sum_{l=1}^{L}\frac{m+1}{r_{l}m}{\left(1-p_{l}\right)}^{2}p_{l}^{2}$. Hence, we have

$$\begin{array}{c} \rho_{0}=cov_{H_{0}}(Z_{\text{SMRT}},Z_{\text{MTT}}(0))=\frac{\sum_{l=1}^{L}\left[p_{l}^{2}{\left(1-p_{l}\right)}^{2}\right]}{\sqrt{[\sum_{l=1}^{L}\frac{1}{r_{l}}p_{l}^{2}{\left(1-p_{l}\right)}^{2}][\sum_{l=1}^{L}r_{l}p_{l}^{2}(1-p_{l}^{2})]}}\\ \rho_{0.5}=cov_{H_{0}}(Z_{\text{SMRT}},Z_{\text{MTT}}(0.5))=0\\ \rho_{1}=cov_{H_{0}}(Z_{\text{SMRT}},Z_{\text{MTT}}(1))=-\frac{\sum_{l=1}^{L}\left[p_{l}^{2}{\left(1-p_{l}\right)}^{2}\right]}{\sqrt{[\sum_{l=1}^{L}\frac{1}{r_{l}}p_{l}^{2}{\left(1-p_{l}\right)}^{2}][\sum_{l=1}^{L}r_{l}(2p_{l}-p_{l}^{2}){\left(1-p_{l}\right)}^{2}]}}. \end{array}$$

Substitute ${\hat{p}}_{l}=[2(r_{2l}+s_{2l})+(r_{1l}+s_{1l})]/[2(m+1)r_{l}]$ for *p_l_*, we obtain the estimate ${\hat{\rho }}_{x}$ for *ρ_x _*(x = 0, 0.5, 1).

Next we report the correlation *ρ_x_*,_0.5 _between *Z*_MTT_(*x*) and *Z*_MTT_(0.5) (x = 0, 1). Under the null hypothesis,

$$\begin{array}{l} \;covH_{0}(U(0),U(0.5))\\ =\sum_{l=1}^{L}\sum_{j=1}^{r_{l}}cov_{H_{0}}\left(\left(mX_{1lj|x=0}-\sum_{k=1}^{m}X_{2ljk|x=0}),\;(mX_{1lj|x=0.5}-\sum_{k=1}^{m}X_{2ljk|x=0.5}\right)\right)\\ =\sum_{l=1}^{L}\sum_{j=1}^{r_{l}}\left[m^{2}cov_{H_{0}}(X_{1lj|x=0},X_{1lj|x=0.5})+\sum_{k=1}^{m}cov_{H_{0}}(X_{2ljk|x=0},X_{2ljk|x=0.5})\right]\\ =(m^{2}+m)\sum_{l=1}^{L}r_{l}(p_{2l}-\frac{1}{2}p_{1l}p_{2l}-p_{2l}^{2}) \end{array}$$

$$\begin{array}{l} \;covH_{0}(U(1),U(0.5))\\ =\sum_{l=1}^{L}\sum_{j=1}^{r_{l}}cov_{H_{0}}\left(\left(mX_{1lj|x=0.5}-\sum_{k=1}^{m}X_{2ljk|x=0.5}),\;(mX_{1lj|x=1}-\sum_{k=1}^{m}X_{2ljk|x=1}\right)\right)\\ =\sum_{l=1}^{L}\sum_{j=1}^{r_{l}}\left\{m^{2}cov_{H_{0}}(X_{1lj|x=0.5},X_{1lj|x=1})+\sum_{k=1}^{m}cov_{H_{0}}(X_{2ljk|x=0.5},X_{2ljk|x=1})\right\}\\ =(m^{2}+m)\sum_{l=1}^{L}r_{l}(\frac{1}{2}p_{1l}+p_{2l}-\frac{1}{2}p_{1l}^{2}-\frac{3}{2}p_{1l}p_{2l}-p_{2l}^{2}). \end{array}$$

Since $V(x)\to m(m+1)\sum_{l=1}^{L}r_{l}(x^{2}p_{1l}+p_{2l}-x^{2}p_{1l}^{2}-p_{2l}^{2}-2xp_{1l}p_{2l})$, after simple algebra, we have,

$$\begin{array}{c} \rho_{0,0.5}=\frac{\sum_{l=1}^{L}r_{l}(p_{2l}-\frac{1}{2}p_{1l}p_{2l}-p_{2l}^{2})}{\sqrt{\left[\sum_{l=1}^{L}r_{l}(p_{2l}-p_{2l}^{2})\right]\left[\sum_{l=1}^{L}r_{l}(\frac{1}{4}p_{1l}+p_{2l}-\frac{1}{4}p_{1l}^{2}-p_{2l}^{2}-p_{1l}p_{2l})\right]}}\\ \rho_{1,0.5}=\frac{\sum_{l=1}^{L}r_{l}\left(\frac{1}{2}p_{1l}+p_{2l}-\frac{1}{2}p_{1l}^{2}-\frac{3}{2}p_{1l}p_{2l}-p_{2l}^{2}\right)}{\sqrt{\left[\sum_{l=1}^{L}r_{l}(p_{1l}+p_{2l}-p_{1l}^{2}-p_{2l}^{2}-2p_{1l}p_{2l})\right]\left[\sum_{l=1}^{L}r_{l}\left(\frac{1}{4}p_{1l}+p_{2l}-\frac{1}{4}p_{1l}^{2}-p_{2l}^{2}-p_{1l}p_{2l}\right)\right]}} \end{array}$$

Substitute ${\hat{p}}_{il}=(r_{il}+s_{il})/[(m+1)r_{l}]$ for *p*_*il *_(i = 0,1,2), we obtain the estimate ${\hat{p}}_{x,0.5}$ for *ρ*_*x*, 0.5 _(x = 0,1).
